# Constraint-based modeling analysis of the metabolism of two *Pelobacter *species

**DOI:** 10.1186/1752-0509-4-174

**Published:** 2010-12-23

**Authors:** Jun Sun, Shelley A Haveman, Olivia Bui, Tom R Fahland, Derek R Lovley

**Affiliations:** 1 Genomatica Inc., 10520 Wateridge Circle, San Diego, CA, USA; 2 Department of Microbiology, University of Massachusetts, Amherst, MA, USA; 3 Luca Technologies Inc., 500 Corporate Circle, Golden, CO, USA; 4 Pathway Genomics Corporation, 4045 Sorrento Valley Blvd., San Diego, CA, USA

## Abstract

**Background:**

*Pelobacter *species are commonly found in a number of subsurface environments, and are unique members of the *Geobacteraceae *family. They are phylogenetically intertwined with both *Geobacter *and *Desulfuromonas *species. *Pelobacter *species likely play important roles in the fermentative degradation of unusual organic matters and syntrophic metabolism in the natural environments, and are of interest for applications in bioremediation and microbial fuel cells.

**Results:**

In order to better understand the physiology of *Pelobacter *species, genome-scale metabolic models for *Pelobacter carbinolicus *and *Pelobacter propionicus *were developed. Model development was greatly aided by the availability of models of the closely related *Geobacter sulfurreducens *and *G. metallireducens*. The reconstructed *P. carbinolicus *model contains 741 genes and 708 reactions, whereas the reconstructed *P. propionicus *model contains 661 genes and 650 reactions. A total of 470 reactions are shared among the two *Pelobacter *models and the two *Geobacter *models. The different reactions between the *Pelobacter *and *Geobacter *models reflect some unique metabolic capabilities such as fermentative growth for both *Pelobacter *species. The reconstructed *Pelobacter *models were validated by simulating published growth conditions including fermentations, hydrogen production in syntrophic co-culture conditions, hydrogen utilization, and Fe(III) reduction. Simulation results matched well with experimental data and indicated the accuracy of the models.

**Conclusions:**

We have developed genome-scale metabolic models of *P. carbinolicus *and *P. propionicus*. These models of *Pelobacter *metabolism can now be incorporated into the growing repertoire of genome scale models of the *Geobacteraceae *family to aid in describing the growth and activity of these organisms in anoxic environments and in the study of their roles and interactions in the subsurface microbial community.

## Background

*Pelobacter *species are commonly found in a number of natural environments, including marine sediments [1], muds [2], soils [3], and hydrocarbon-containing environments [4-6]. They likely play important roles in syntrophic degradation of organic matter in these natural environments and are of interest for applications in bioremediation and microbial fuel cells. *Pelobacter *species and members of *Geobacter*, *Desulfuromonas *and *Desulfuromusa *genera in the deltaproteobacteria form the monophyletic family *Geobacteraceae*, which can be divided into two distinct subgroups of the *Geobacter *and *Desulfuromonas *clusters [7-9]. The *Pelobacter *species are phylogenetically intertwined with both clusters: *Pelobacter carbinolicus *is within the *Desulfuromonas *cluster whereas *Pelobacter propionicus *is within the *Geobacter *cluster [7,9,10].

*P. carbinolicus *and *P. propionicus *are both strict anaerobes that have distinct physiologies compared with the *Geobacter *and *Desulfuromonas *species. *P. carbinolicus *was first isolated from marine muds whereas *P. propionicus *was first isolated from freshwater sediments and sewage sludge [6]. Both can grow by fermentation of 2,3-butanediol and acetoin in which the fermentation products are ethanol and acetate for *P. carbinolicus*, and propionate and acetate for *P. propionicus *[6]. In addition, both *Pelobacter *species can grow with ethanol, propanol, or butanol under specific conditions [6].

Like *Geobacter *and *Desulfuromonas *species, *Pelobacter *species have the capacity of using S^0 ^as an electron acceptor [7,10]. However, *Pelobacter *species only incompletely oxidize organic substrates with this electron acceptor, in contrast to the ability of *Geobacter *and *Desulfuromonas *species to completely oxidize acetate and other organic electron donors to carbon dioxide [7,10,11]. For example, *P. carbinolicus *only incompletely oxidizes ethanol to acetate with S^0 ^reduction [10].

Unlike *Geobacter *and *Desulfuromonas *species, *P. carbinolicus *lacks most of the *c*-type cytochromes [12], which are essential for optimal electron transfer to Fe(III) in *Geobacter sulfurreducens *[13-16]. *P. carbinolicus *reduces Fe(III) via an indirect mechanism in which elemental sulfur is reduced to sulfide and the sulfide reduces Fe(III) with the regeneration of elemental sulfur, contrasting with the direct reduction of Fe(III) for *Geobacter *species [17]. Furthermore, whereas the *Geobacter *and *Desulfuromonas *species can transfer electrons to the anodes of microbial fuel cells to produce current, *P. carbinolicus *could not [18]. Thus, the *Pelobacter *species are regarded as primarily fermentative/syntrophic species, whereas the *Geobacter *and *Desulfuromonas *species are primarily respiratory species. Comparative genomic studies have suggested that the common ancestor of *Pelobacter, Geobacter*, and *Desulfuromonas *species was a respiratory microorganism and that *Pelobacter *species evolved to fill fermentative and syntrophic niches (4).

*Pelobacter *and *Geobacter *species can be closely associated in subsurface environments [19,20]. In microbial fuel cells, a coculture of *P. carbinolicus *and *G. sulfurreducens *produced current with ethanol as the fuel, under conditions in which current could not be produced by either microorganism alone [18]. Genome-based experimental and computational techniques offer the possibility of predictive modeling microbial physiology and microbial communities [21]. Genome-based analysis of *Geobacter *species resulted in two constraint-based genome-scale metabolic models [22,23], which were applied in many studies to accelerate our understanding of *Geobacter *species and their applications [24-29]. The genome sequence of *P. carbinolicus *DSM2380 and *P. propionicus *DSM2379 have been completed http://www.jgi.doe.gov. Because of the similarities and differences in metabolic capabilities between *Pelobacter *and *Geobacter *species, *in silico *modeling of these two *Pelobacter *species will provide insight into their metabolism. In this report, the developments of genome-scale metabolic models of *P. carbinolicus *and *P. propionicus *are described. These models of *Pelobacter *metabolism can now be incorporated into the growing repertoire of genome scale models of the *Geobacteraceae *family to aid in describing the growth and activity of these organisms in anoxic environments and in the study of their roles and interactions in the subsurface microbial community.

## Methods

### Strains and culture conditions

*P. carbinolicus *DSM 2380 was cultured at 30°C under strictly anaerobic conditions as previously described [12]. Fermentative cultures were grown in chemostats in bioreactor at dilution rates of 0.03 to 0.06 h^-1 ^with 5 mM acetoin in the medium. Cell growth on acetoin was monitored by measuring the optical density at 600 nm with a Genesys 2 spectrophotometer (Spectronic Instruments, Rochester, NY). Cell dry weight was determined gravimetrically after drying at 105°C for 24 h.

### Analytical measurements

Concentrations of acetoin, acetate, and ethanol were determined with high-pressure liquid chromatography (HPLC) using an LC-10ATVP HPLC (Shimadzu, Kyoto, Japan) equipped with an Aminex HPX-87 H column (300 by 7.8 mm; Bio-Rad, Hercules, CA), with 8 mM H_2_SO_4 _eluent. Acetate was detected with an SPD-10VP UV detector (Shimadzu, Kyoto, Japan) set at 210 nm. Ethanol and acetoin were quantified with an RID-10A refractive index detector (Shimadzu, Kyoto, Japan). Protein concentrations were determined with the bicinchoninic acid (BCA) method [30] with bovine serum albumin as standard.

### Metabolic network reconstruction

The *Pelobacter *metabolic networks were reconstructed according to previously published procedures [23]. The reconstruction was carried out in SimPheny [31,32] (Genomatica, Inc., CA) from the annotated open reading frames (ORFs) encoded in the *Pelobacter *genomes. The *Pelobacter *genomes and the genomes of several high-quality genome-scale metabolic models were analyzed using the sequence similarity search (BLAST), and the BLAST results were utilized to create draft models that served to accelerate the reconstruction of the genome-scale metabolic models. The reactions and genes in the draft models were manually reviewed using the gene annotations and the available biochemical and physiological information. Biomass compositions in the published *G. sulfurreducens *model were used to create the biomass demand reactions in both reconstructed *Pelobacter *models. The resulting networks were then subjected to the gap filling process to allow biomass formation under physiological growth conditions. For gap filling, simulations were performed to determine if the networks could synthesize every biomass component and the missing reactions in the pathways were identified. These reactions were reviewed for gene association, or added as non-gene associated reactions to enable the formation of biomass by the reconstructed networks under physiological conditions. Experimental data were collected from published literature or generated in the laboratory, and were applied to validate model simulation results that predict growth and products under corresponding physiological conditions. Model simulations were also used to generate experimentally testable hypotheses and predictions. The experimental findings accordingly were in turn used to further refine and expand the metabolic models in an iterative process.

The detailed list of genes, reactions, metabolites, and gene-protein-reaction (GPR) associations in the metabolic model are available as additional files.

### Determination of energy parameters of the metabolic model

Energy parameters of the *P. carbinolicus *metabolic model including growth-associated maintenance (GAM) energy and non-growth associated maintenance (nGAM) energy were determined using four sets of experimental data obtained in chemostats of *P. carbinolicus *during acetoin fermentation at dilution rates of 0.03 to 0.06 h^-1^. The experimental data including acetoin uptake rates and acetate production rates were used as constraints to simulate fermentative growth where the ATP maintenance requirement reaction was selected as the objective function to be maximized. The maximum ATP production rates were calculated and plotted against the dilution rates as described earlier [33]. The linear regression of the plot resulted in an equation:

$${\text{q}}_{\text{ATP}}=a\times \mu +b$$

where **q**_ATP _is the ATP production rate and **μ **is the dilution rate. The intercept ***b ***represents nGAM, the ATP requirement at zero growth, whereas the slope ***a ***represents GAM, the ATP requirement for growth. GAM was then incorporated into the biomass demand equation, and nGAM was utilized for all further growth simulations.

For the *P. propionicus *metabolic model, the same biomass demand equation including GAM was applied. The nGAM was estimated using *P. propionicus *fermentative growth yields with 6 different substrates [6].

### *In silico *analysis of metabolism

The metabolic capabilities of the *Pelobacter *models were calculated using flux balance analysis through linear optimization [34] in SimPheny. The simulations resulted in flux values in units of mmol/g dry weight (gdw)/h. All simulations were of anaerobic growth on minimal media, where the following external metabolites were allowed to freely enter and leave the network: CO_2_, H^+^, H_2_O, K^+^, Mg^2+^, NH_4_^+^, PO_4_^3-^, and SO_4_^2-^. The electron donors or electron acceptors tested were allowed a maximum uptake rate into the network as specified in the results. All other external metabolites were only allowed to leave the system. To simulate physiological conditions, experimental data were used as constraints for flux optimization. Growth simulations were carried out for: 1) optimal growth where biomass synthesis was selected as the objective function to be maximized with substrate uptake rate fixed; or 2) optimal substrate utilization where the substrate uptake rate was chosen as the objective function to be minimized with the growth rate fixed.

## Results and Discussion

### Metabolic network reconstruction

The *P. carbinolicus *draft model was built with base models including previously published *Geobacter sulfurreducens *[22], *Escherichia coli *[34], and *Bacillus subtilis *[35] models. The *P. propionicus *draft model was prepared with the above base models plus the completed *P. carbinolicus *and *Geobacter metallireducens *[23] models. The origin of reactions in the draft models closely reflected the phylogenetic relationships among the species [7]. For example, reactions in the *P. propionicus *draft model originating from the *G. sulfurreducens*, *G. metallireducens*, and *P. carbinolicus *models were 40%, 31%, and 14%, respectively, consistent with the fact that *P. propionicus *is more closely related to *G. sulfurreducens *and *G. metallireducens *than to *P. carbinolicus *[7].

The reactions and their gene associations in the draft models of *P. carbinolicus *and *P. propionicus *were evaluated manually based on gene annotations, published biochemical and physiological information and external references as previously described [23]. One important metabolic characteristic of *Pelobacter *species is their fermentative growth on acetoin [6]. During acetoin fermentation, acetoin is degraded into acetaldehyde and acetyl-CoA by the acetoin:2,6-dichlorophenolindophenol (DCPIP) oxidoreductase [36,37]. The acetoin:DCPIP oxidoreductase was purified and characterized in *P. carbinolicus *[38] and was encoded by the *acoA*, *acoB*, *acoC*, and *acoL *genes [37]. In the reconstructed *Pelobacter *networks, the reaction was associated with the *acoA *(Pcar_0343 & Ppro_1131), *acoB *(Pcar_0344 & Ppro_1132), *acoC *(Pcar_0345 & Ppro_1133), and *acoL *(Pcar_0347 & Ppro_1137) genes. In the *P. carbinolicus *model, the resulting acetaldehyde is reduced to ethanol by alcohol dehydrogenase, whereas acetyl-CoA is converted into acetate by phosphate acetyltransferase and acetate kinase (Pcar_2542 & Pcar_2543) generating ATP, as described previously [36]. In the *P. propionicus *model, acetaldehyde is oxidized to acetyl-CoA by acetaldehyde CoA dehydrogenase (Ppro_0899 or Ppro_1923), and acetyl-CoA is then converted into propionate in a pathway through pyruvate, oxaloacetate, malate, fumarate, succinate, succinyl-CoA, methylmalonyl-CoA, and propionyl-CoA [39]. The key enzyme in this pathway, methylmalonyl-CoA mutase, was associated with genes Ppro_1367 & Ppro_1368. Reactions and their associated genes for fermentative growth on other substrates were also evaluated. Transportation of neutral molecules such as acetoin, 2,3-butanediol, and other alcohols were assumed to be energy free through reversible diffusion.

*P. propionicus *ferments both ethanol and lactate to acetate and propionate at an approximately 1:2 ratio via methylmalonyl-CoA, but the molar biomass yield with ethanol was less than half of that obtained with lactate [6,40]. It was suggested that the different biomass yields were likely due to either a metabolic energy requirement for the ferredoxin-dependent reductive carboxylation of acetyl-CoA to pyruvate, or energy conservation in lactate uptake [40]. Since the metabolic energy requirement for acetyl-CoA carboxylation was not demonstrated with any *Geobacteraceae *family member [22,23], a lactate proton antiporter was utilized to allow energy conservation in lactate uptake in the reconstructed *P. propionicus *network.

*Pelobacter *species have been reported to use Fe(III) or S^0 ^as an electron acceptor [7,10], but studies with *P. carbinolicus *have suggested that Fe(III) is reduced indirectly through sulfur reduction, with sulfide serving as an electron shuttle for the reduction of Fe(III) [17]. It was suggested that a low concentration of sulfide favored Fe(III) reduction without precipitation of ferrous mono- or disulfides in *Sulfurospirillum deleyianum *[41]. Given the standard reduction potentials for the Fe^3+^/Fe^2+ ^pair (E'° = +771 mV) and the S^0^/H_2_S pair (E'° = -243 mV) [42], an extracellular nonenzymatic reaction FE3Rs (2 Fe^3+ ^+ HS^- ^→ 2 Fe^2+ ^+ S + H^+^) was used to model the indirect Fe(III) reduction. In other bacteria, sulfide is regenerated from elemental sulfur by the membrane-bound sulfur reductase that contains both hydrogenase and sulfur reductase activities [43,44]. The sulfur reductase can use hydrogen as electron donor, or NAD(P)H at a reduced activity [43]. Thus, two sulfur reductase reactions SRE and SRE2 were utilized in the reconstructed *P. carbinolicus *network for sulfur reduction with hydrogen or NAD(P)H as electron donors, although the enzymes catalyzing Fe(III) and S^o ^reduction in *P. carbinolicus *have not been identified experimentally.

The gap filling process identified some missing reactions in the pathways that were added as non-gene associated reactions to enable the reconstructed network to synthesize metabolites for biomass formation. The reconstructed *P. carbinolicus *network contains 37 non-gene associated reactions whereas the *P. propionicus *network contains 46 non-gene associated reactions. These non-gene associated reactions are presumptive metabolic functions encoded potentially by unknown genes, and thus will be the subject of further genomic and biochemical investigation in the future.

Recently, a sensitivity analysis in *E. coli *model [45] indicated that small changes in biomass compositions such as protein, RNA, and lipid did not significantly affect predicted growth rates. Biomass compositions in the published *G. sulfurreducens *model [22] were used to create the biomass demand reactions in both reconstructed *Pelobacter *models, as they are closely related to *G. sulfurreducens*. Sensitivity analysis also indicated minimal changes in simulations by small biomass composition changes in the *Pelobacter *models (data not shown). Metabolic maps including the biomass demand reactions for the *P. carbinolicus *and *P. propionicus *genome-scale metabolic models were provided (see Additional File 1).

### Determination of energy parameters and stoichiometry of the electron transport chain

**Figure 1:**
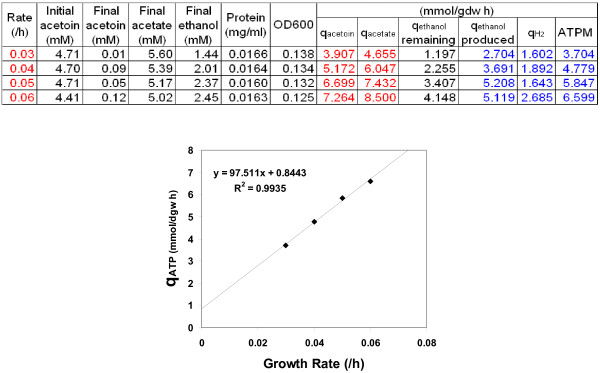
**Determination of the energy parameters of the *P. carbinolicus *metabolic model**. The table shows four sets of physiological data at different dilution rates for *P. carbinolicus *during acetoin fermentation in chemostats. The *red *experimental data were applied as constraints in simulations to obtain the predicted *blue *values, which were then used to generate the linear correlation graph to determine energy parameters of the *P. carbinolicus *metabolic model.

The energy parameters of the *P. carbinolicus *metabolic model were determined using four sets of physiological data from *P. carbinolicus *during acetoin fermentation in chemostats producing acetate and ethanol. The gassing required for strict anaerobic growth conditions in chemostats could result in: 1) partial loss of ethanol produced from acetoin fermentation through evaporation; 2) ethanol fermentation to produce acetate and H_2_, where H_2 _was removed by gassing to release the feedback inhibition. Therefore, only the experimental acetoin uptake rates and acetate production rates were used to simulate the fermentative growth (Figure 1). The linear correlation of **q**_ATP _= 97.5 × **μ **+ 0.844 has a R^2 ^= 0.994 indicating high quality of the results. Therefore, the GAM for the *P. carbinolicus *model is 97.5 mmol ATP/gdw and the nGAM is 0.844 mmol ATP/gdw/h.

To determine energy parameters of the *P. propionicus *metabolic model, GAMs from the *P. carbinolicus *model and the *G. sulfurreducens *model [22] were used to simulate experimental results. The *P. propionicus *2,3-butanediol fermentation had an optimal growth rate **μ **= 0.144 h^-1 ^[6] that was used for simulating *P. propionicus *fermentative growth with 2,3-butanediol, acetoin, and lactate due to similar growth yields for these fermentations. The growth rates and calculated substrate fluxes based on experimental data [6] were used as constraints in simulations to optimize ATP production. The average of the ATP production rates, representing the nGAM of the *P. propionicus *model, was 2.80 mmol ATP/gdw/h calculated using the GAM of the *P. carbinolicus *model. Therefore, the GAM for the *P. propionicus *model is 97.5 mmol ATP/gdw and the nGAM is 2.80 mmol ATP/gdw/h.

**Figure 2:**
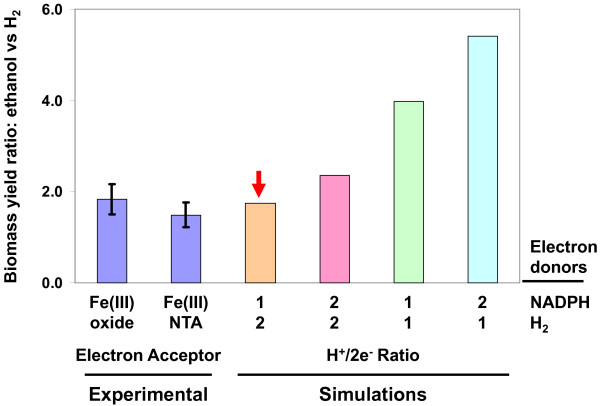
**Determination of the stoichiometry of electron transfer in the two sulfur reductase reactions**. The relative biomass yield ratios of ethanol to hydrogen were obtained from experimental data of *P. carbinolicus *grown in batch culture with ethanol or hydrogen as electron donor and Fe(III) oxide or Fe(III)-NTA as electron acceptor [17], and were compared to simulation results varying the stoichiometry of the electron transfer in the two sulfur reductase reactions. The *red arrow *indicates a close match between experimental and simulation results.

The *Pelobacter *models contain two sulfur reductase reactions SRE and SRE2 with hydrogen or NADPH as electron donors. To determine the stoichiometry of the proton translocation per pair of electrons transferred (H^+^/2e^-^) in the SRE and SRE2 two reactions, the published biomass yield data for *P. carbinolicus *grown in batch culture with ethanol or hydrogen as electron donor and Fe(III) as electron acceptor [17] were utilized. The biomass yield with ethanol as electron donor was compared to the biomass yield with hydrogen as electron donor, and the biomass yield ratios calculated from experimental results were compared to those from simulation results, where the H^+^/2e^- ^ratio varied from 1 to 2 for SRE with hydrogen as electron donor or for SRE2 with ethanol as electron donor (Figure 2). As shown in Figure 2, simulation results with the H^+^/2e^- ^ratio of 2 for SRE and the H^+^/2e^- ^ratio of 1 for SRE2 closely matched experimental results, whereas the other three combinations resulted in unmatched biomass yield ratios. Thus, the H^+^/2e^- ^ratios were determined as 2 for SRE and 1 for SRE2 in *P. carbinolicus *model.

### Metabolic models of *P. carbinolicus *and *P. propionicus*

Upon completion, the genome-scale *P. carbinolicus *metabolic model included 741 genes of the 3389 genes in the *P. carbinolicus *genome. As shown in Table 1, the *P. carbinolicus *model contains 708 reactions and 765 metabolites including 70 extracellular metabolites (see Additional File 2 for list of genes, reactions, and metabolites included in the *P. carbinolicus *metabolic model).

**Table 1:** Characteristics of the P. carbinolicus and P. propionicus genome-scale metabolic models compared with those of the G. sulfurreducens and G. metallireducens models.

	*P. carbinolicus*		*P. propionicus*		*G. sulfurreducens*		*G. metallireducens*	
**Total Genes**	3389		3831		3468		3532	
Included Genes	741	(21.9%)	661	(17.3%)	712	(20.5%)	747	(21.1%)
Excluded Genes	2648	(78.1%)	3170	(82.7%)	2756	(79.5%)	2785	(78.9%)
								
**Total Proteins**	625		532		573		623	
								
**Total Reactions**	708		650		650		697	
Non-gene Reactions	37	(5.2%)	46	(7.1%)	34	(5.2%)	30	(4.3%)
								
**Total Exchange Reactions**	69		53		58		61	
								
**Total Metabolites**	765		708		701		769	
Extracellular Metabolites	70	(9.2%)	52	(7.3%)	57	(8.1%)	60	(7.8%)

The genome-scale *P. propionicus *metabolic model included 661 genes among the 3831 genes in the *P. propionicus *genome. As shown in Table 1, the *P. propionicus *model consists of 650 reactions and 708 metabolites including 52 extracellular metabolites (see Additional File 3 for list of genes, reactions, and metabolites included in the *P. propionicus *metabolic model).

As shown in Table 1, the *Pelobacter *models included about 20% of the genes in their genomes, similar to the *Geobacter *models [22,23]. The numbers of total reactions in the *Pelobacter *and *Geobacter *models are also comparable. Thus, these *Geobacteraceae *family members contain similar size genomes, share similar basic metabolic functions, and result in similar size genome-scale metabolic models.

**Figure 3:**
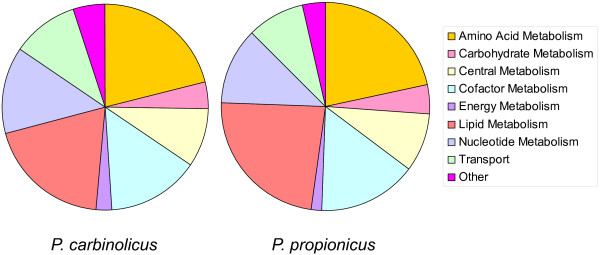
**Functional classifications of reactions in the two *Pelobacter *metabolic models**. Reactions in each *Pelobacter *metabolic model were categorized into 9 functional classifications.

The reactions in both *Pelobacter *metabolic models were categorized according to 9 functional classifications (Figure 3). The two models share a similar distribution of reactions among different functional groups: reactions for metabolism of amino acids, lipids, cofactors, and nucleotides are the most abundant, accounting for almost 70% of all the reactions in both *Pelobacter *models.

Currently, there are 76 reactions associated with transporting metabolites in the *P. carbinolicus *model and 57 reactions for transporting metabolites in *P. propionicus *model, including redundant transporters for some extracellular metabolites. In addition, both *Pelobacter *genomes contained many genes related to ABC transport systems, which were not included in the network due to the lack of substrate specificity. Future experiments could provide additional evidence to include more transport systems.

### Validation of the metabolic models using published experimental data

The *Pelobacter *metabolic models were validated using published experimental growth data. In cases where the experimental data was obtained from batch culture experiments where flux data was not readily available, experimentally determined growth rates were used to constrain the simulations. Ratios of biomass, substrate and product fluxes from model simulations were calculated and compared with the ratios from experimental value.

**Figure 4:**
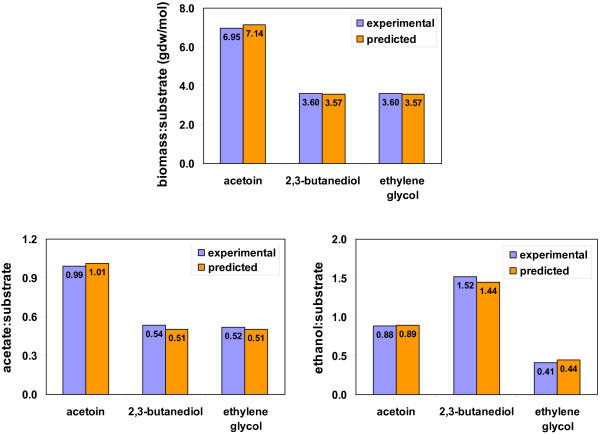
**Validation of the *P. carbinolicus *metabolic model for fermentative growth**. The biomass yield per mol of substrate, the ratios of produced acetate to substrates, and the ratios of produced ethanol to substrates were obtained from experimental data of *P. carbinolicus *fermenting acetoin, 2,3-butanediol, and ethylene glycol [6], and were compared to those predicted by *in silico *simulations with the *P. carbinolicus *metabolic model.

*P. carbinolicus *can grow by fermenting acetoin, 2,3-butanediol, and ethylene glycol [6]. The *P. carbinolicus *metabolic model was validated by comparing experimental results with predicted results from *in silico *simulations of *P. carbinolicus *fermentative growth on these substrates (Figure 4). An optimal growth rate of 0.087 h^-1 ^was observed with *P. carbinolicus *fermentative growth on 2,3-butanediol [6], and was applied to constrain all three simulations in which the substrate uptake rate was minimized. In simulations, the substrates acetoin, 2,3-butanediol, and ethylene glycol were consumed to produce acetate and ethanol. The biomass yield per mole of acetoin was twice the biomass yield per mole of 2,3-butanediol and ethylene glycol in simulations, which matched well with the experimental data [6]. Similarly, the ratios of produced acetate and ethanol to consumed substrates predicted by *in silico *simulations were validated by the ratios from experimental results [6]. Thus, the fermentative growth of the *P. carbinolicus *metabolic model has been validated and can be applied in future modeling studies.

**Figure 5:**
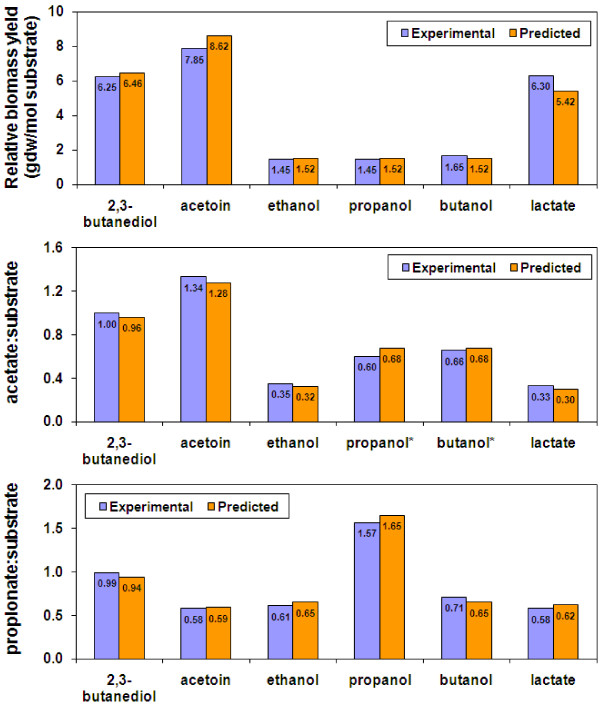
**Validation of the *P. propionicus *metabolic model for fermentative growth**. The biomass yield per mol of substrate, the ratios of acetate to substrates, and the ratios of produced propionate to substrates were obtained from experimental data of *P. propionicus *fermenting 2,3-butanediol, acetoin, ethanol, propanol, butanol, and lactate [6], and were compared to those predicted by *in silico *simulations with the *P. propionicus *metabolic model. * indicating that acetate was consumed during fermentation of propanol and butanol.

Similarly, the fermentative growth of the *P. propionicus *metabolic model was validated with experimental data obtained from *P. propionicus *fermentation of acetoin, 2,3-butanediol, ethanol, propanol, butanol, and lactate [6]. An optimal growth rate of 0.144 h^-1 ^was observed with 2,3-butanediol fermentative growth [6], and was applied to constrain the simulations in which the substrate uptake rate was minimized. The predicted results from *in silico *simulations were compared with experimental results (Figure 5). In simulations, acetoin, 2,3-butanediol, lactate, and ethanol were consumed to produce acetate and propionate, whereas substrates and acetate were both consumed to produce propionate in propanol and butanol fermentations. As shown in Figure 5, the biomass yield per mole of acetoin was higher than the biomass yields per mole of 2,3-butanediol and lactate in simulations, and the latter two were much higher than the biomass yields per mole of the alcohols. The predicted biomass yields and ratios of acetate:substrate or propionate:substrate matched well with the experimental results. Therefore, the *P. propionicus *metabolic model has been validated with experimental fermentative growth results. In the *P. propionicus *metabolic model, lactate uptake was assumed to occur through a lactate proton antiporter for energy conservation and the simulated results predicted about 14% less biomass yield than the experimental results. Other lactate transporters such as a lactate proton symporter or diffusion were tested in simulations and resulted in much larger discrepancies with 38%-63% less biomass yields than the experimental results, supporting the assignment of a lactate proton antiporter for lactate uptake. However, further experimentation is required to confirm how lactate is transported.

*P. carbinolicus *can ferment ethanol to acetate with hydrogen generation in coculture with either *Acetobacterium woodii *or *Methanospirillum hungatei *as hydrogen utilizing partners [6]. Simulations of *P. carbinolicus *growth on 2,3-butanediol and ethanol with hydrogen production were conducted. The simulations predicted ratios of produced acetate to consumed substrates at 1.80 for acetate:2,3-butanediol and at 0.90 for acetate:ethanol, matched well with the experimental results at 1.96 for acetate:2,3-butanediol and at 0.93 for acetate:ethanol. The simulation results predicted a growth yield ratio of 2.0 for 2,3-butanediol to ethanol, close to the experimental growth yield ratio of 2.2 for 2,3-butanediol to ethanol. The *P. carbinolicus *model was validated for the hydrogen production for syntrophic growth with experimental results.

**Figure 6:**
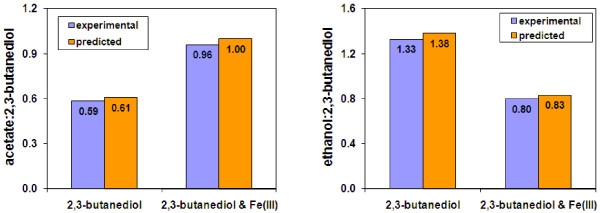
**Validation of the *P. carbinolicus *metabolic model for growth with Fe(III) reduction**. The ratios of produced acetate and ethanol to 2,3-butanediol were obtained from experimental data of *P. carbinolicus *grown on 2,3-butanediol with or without Fe(III) [10], and were compared to those predicted by *in silico *simulations with the *P. carbinolicus *metabolic model.

Like phylogenetically related *Geobacter *and *Desulfuromonas *species within the family *Geobacteraceae*, *P. carbinolicus *can utilize Fe(III) as the terminal electron acceptor [10], but sulfide is likely to serve as an electron shuttle for the Fe(III) reduction in *P. carbinolicus *[17]. *P. carbinolicus *grown on 2,3-butanediol with Fe(III) produced less ethanol and more acetate than grown on 2,3-butanediol without Fe(III) [10]. The experimental acetate:ethanol ratios and growth rates were applied as constraints to simulate Fe(III) reduction by optimizing 2,3-butanediol utilization. Simulation results of *P. carbinolicus *growth on 2,3-butanediol with or without Fe(III) are shown in Figure 6. The ratios of ethanol:2,3-butanediol and acetate:2,3-butanediol in the presence or absence of Fe(III) from *in silico *simulations matched well with those from the experimental data [10]. These results indicated that *P. carbinolicus *model worked well with Fe(III) reduction simulations.

**Figure 7:**
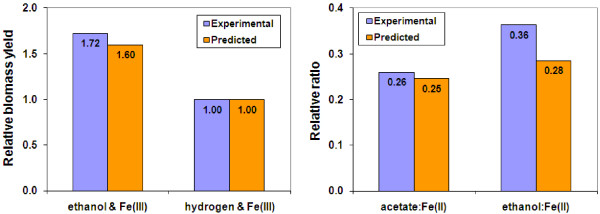
**Validation of the *P. carbinolicus *metabolic model for growth utilizing hydrogen coupled with Fe(III) reduction**. The relative biomass yield of ethanol to hydrogen and the ratios of acetate and ethanol to Fe(II) produced were obtained from experimental data of *P. carbinolicus *growth on hydrogen/acetate or ethanol with Fe(III) as the electron acceptor [10], and were compared to those predicted by *in silico *simulations with the *P. carbinolicus *metabolic model.

*P. carbinolicus *is capable of utilizing H_2 _as the electron donor coupled with Fe(III) reduction to support growth when acetate is provided as the carbon source [10]. *P. carbinolicus *growth on ethanol or on hydrogen with acetate as the carbon source and with Fe(III) as the electron acceptor was simulated by applying experimental growth rates as constraints and optimizing the substrate utilization. As shown in Figure 7, the biomass yield ratios between ethanol/Fe(III) and H_2_/Fe(III) growth were calculated from both *in silico *result and experimental data, and were closely matched. Additionally, the relative ratios of acetate produced to Fe(III) reduced and ethanol consumed to Fe(III) reduced under ethanol/Fe(III) growth conditions were similar between predicted and experimental results. The stoichiometry of Fe(III) reduced to hydrogen consumed was measured as 1.9 in experiments [10] and was calculated as 1.83 in simulations. These results were consistent between predicted and experimental values, and validated the *P. carbinolicus *model for H_2 _and ethanol utilization coupled with Fe(III) reduction.

### Comparison of the four *Geobacteraceae *metabolic models

The four *Geobacteraceae *metabolic models were compared at the reaction level. The common reactions in all 4 models were determined for each functional category and the unique reactions in only one model but not in the other models were also determined (Table 2). The functional distribution of reactions among the four metabolic models shows similar patterns, with amino acid and lipid metabolism accounting for the most reactions and the energy metabolism category comprising the least reactions. Overall, 470 common reactions are present in all 4 models. These common reactions are largely distributed in functional categories including amino acid metabolism, cofactor metabolism, lipid metabolism, and nucleotide metabolism. Among the common reactions, the complete TCA cycle reactions are present in all 4 models, yet the *Pelobacter *species can not completely oxidize acetate and other organic electron donors to carbon dioxide [10] while the *Geobacter *species can. Another common reaction that is present in all 4 models is the nitrogenase reaction. The molybdenum nitrogenase enzyme complex contains the Fe protein encoded by the *nifH *gene and the MoFe protein encoded by the *nifD *and *nifK *genes [46,47], and the genes are well conserved among the four *Geobacteraceae *family members. The presence of the nitrogenase complex in all metabolic models suggested that all 4 species can fix nitrogen through ATP-dependent reduction of nitrogen to ammonia for growth, and it has shown that *G. sulfurreducens *can grow by nitrogen fixation [48].

**Table 2:** Comparison of reactions in the P. carbinolicus, P. propionicus, G. sulfurreducens, and G. metallireducens metabolic models.

	*P. carbinolicus*			*P. propionicus*			*G. sulfurreducens*			*G. metallireducens*			Common
		
	All	Unique	%	All	Unique	%	All	Unique	%	All	Unique	%	
Amino Acid Metabolism	149	13	8.7%	141	4	2.8%	149	3	2.0%	143	4	2.8%	118

Carbohydrate Metabolism	30	5	16.7%	28	0	0.0%	21	0	0.0%	24	5	20.8%	13

Central Metabolism	65	6	9.2%	60	6	10.0%	56	1	1.8%	62	5	8.1%	39

Cofactor Metabolism	103	3	2.9%	99	2	2.0%	106	1	0.9%	104	0	0.0%	93

Energy Metabolism	16	6	37.5%	11	1	9.1%	22	4	18.2%	20	1	5.0%	8

Lipid Metabolism	138	8	5.8%	153	2	1.3%	130	4	3.1%	159	18	11.3%	98

Nucleotide Metabolism	96	24	25.0%	77	0	0.0%	77	0	0.0%	76	2	2.6%	65

Transport	76	19	25.0%	57	1	1.8%	62	10	16.1%	62	22	35.5%	21

Other	35	8	22.9%	24	1	4.2%	27	2	7.4%	47	22	46.8%	15

													

Total reactions	708	92	13.0%	650	17	2.6%	650	25	3.8%	697	79	11.3%	470

Although the four species shared many common reactions among their metabolic models, each model contains some unique reactions, reflecting the unique metabolic capabilities of the *Geobacteraceae *species. As shown in Table 2, the *P. carbinolicus *model contains 92 unique reactions and the *G. metallireducens *model contains 79 unique reactions, whereas the *P. propionicus *model contains only 17 unique reactions and the *G. sulfurreducens *model contains 25 unique reactions. For the *P. carbinolicus *model, these unique reactions include, for example: ethylene glycol dehydratase for the fermentation of ethylene glycol; purine-nucleoside phosphorylase for the formation of purines like adenosine, deoxyadenosine, xanthosine, and hypoxanthine; and acetylornithine deacetylase and ornithine cyclodeaminase to form a second pathway for proline biosynthesis through L-ornithine in addition to the common proline biosynthesis pathway through 1-pyrroline-5-carboxylate. Additionally, *P. carbinolicus *contains the pyruvate formate lyase and formate dehydrogenase reactions that can convert pyruvate to acetyl-CoA with the co-production of hydrogen for syntrophic growth with hydrogen-consuming organisms. The unique reactions in the *P. propionicus *model include the reactions for the methylmalonyl-CoA dependent propionate formation pathway. The citrate lyase reaction is also unique to the *P. propionicus *model but its physiological role is not clear yet. See Additional File 4 for a list of unique reactions in the four *Geobacteraceae *metabolic models.

### Incorporation of gene expression levels in metabolic models

The reconstructed metabolic models contain duplicate genes for the same reaction, or alternative reactions between two metabolites, or alternative pathways among metabolites. Without additional constraints, the duplicate genes, alternative reactions, or alternative pathways can all direct metabolic fluxes in simulations. Gene expression data suggesting the preference of duplicate genes, alternative reactions, or alternative pathways can be utilized to constrain the simulations and improve computational modeling analysis. On the other hand, the metabolic models can also be utilized to facilitate the interpretation of gene expression data. The *P. carbinolicus *metabolic model and the microarray data obtained from *P. carbinolicus *cells cultured by acetoin fermentation or ethanol/Fe(III) respiration [17] were compared.

During acetoin fermentation, acetoin is degraded into acetaldehyde and acetyl-CoA by the acetoin:DCPIP oxidoreductase. When the gene expression levels were displayed on the *P. carbinolicus *metabolic map, genes for the acetoin:DCPIP oxidoreductase Pcar_0343-0347 were among the most highly transcribed genes during acetoin fermentation. During ethanol/Fe(III) respiration, the expression levels of these genes decreased more than 10 fold [17]. Genes with the highest transcription levels during ethanol/Fe(III) respiration include Pcar_0251 and Pcar_0255 for the alcohol dehydrogenase (ethanol:NAD) reaction and Pcar_2758 for the acetaldehyde dehydrogenase reaction, involved in the oxidation of ethanol to acetyl-CoA[17]. These genes were downregulated more than 5 fold during acetoin fermentation compared to ethanol/Fe(III) respiration [17]. The metabolic map and model added an additional tool to understand the gene expression data from a metabolic point of view.

The metabolic model contains redundancy in the form of duplicate genes, alternative reactions, or alternative pathways. Such redundancy may play important roles in genetic and metabolic robustness. Microarray analysis indicated that this redundancy was tightly regulated in *P. carbinolicus*. For example, two acetyl-CoA:phosphate acetyltransferase isozymes Pcar_2542 and Pcar_2850 are present in the *P. carbinolicus *model. The gene expression level of Pcar_2542 was 23 fold more than the level of Pcar_2850 during acetoin fermentation, suggesting that Pcar_2542 was the primary enzyme to catalyze the reaction in acetoin fermentation. Similar examples included isozymes for aconitase, acetate kinase, acetoin dehydrogenase, acetaldehyde dehydrogenase, etc.

When different reactions for the same metabolite are present, it is likely for the cell to use the most energy efficient reaction for optimal growth. An example of this is the presence of a sulfate proton symport transporter and a sulfate ABC transport system for sulfate transport. The expression level of the proton symport transporter gene Pcar_0676 was more than the sulfate ABC transport system genes during acetoin fermentation (expression of Pcar_0676 was about 8 times Pcar_2084). Thus, it was likely that sulfate was mainly transported through the proton symport transporter during acetoin fermentation. Similarly, the genes encoding nitrogen fixation enzymes were turned off as the cell used ammonium from the culture medium. The *P. carbinolicus *metabolic model contains a biosynthesis pathway for proline from glutamate through ornithine, in addition to the 1-pyrroline-5-carboxylate-dependent pathway present in the *G. sulfurreducens *model. Microarray results indicated that the mRNA levels of pyrroline-5-carboxylate reductase and ornithine cyclodeaminase were similar under both acetoin fermentation and ethanol/Fe(III) respiration conditions, suggesting that both pathways were used for proline biosynthesis.

*P. carbinolicus *contains genes for *de novo *cobalamin biosynthesis, but some of these genes were among the lowest expression levels during both culture conditions. Most likely, *P. carbinolicus *did not synthesize cobalamin, but utilized the vitamin B_12 _supplemented in the culture medium. When compared to the *G. sulfurreducens *model, *P. carbinolicus *had some unique reactions allowing the formation of purines like adenosine, inosine, deoxyinosine, xanthine, hypoxanthine, and urate. However, gene expression data suggested that only adenosine was formed during both culture conditions, whereas the others were unlikely to be synthesized.

The microarray results will be utilized to constrain the reconstructed network, where the reactions are closed during simulations when the associated genes have very low expression level for the corresponding culture conditions.

### TCA cycle and sulfur reduction

*P. carbinolicus *reduces Fe(III) indirectly through sulfur reduction [17]. However, *P. carbinolicus *only incompletely oxidizes organic substrates to acetate using Fe(III) as an electron acceptor [10]. The complete TCA cycle reactions are present in *P. carbinolicus *model, yet the fully functional TCA cycle was not observed during simulations with Fe(III) as electron acceptor. Further examination suggested that this was due to the inability in coupling succinate oxidation to sulfur reduction (ΔG_0_' = +53 kJ/mol) [49]. Many archaea and bacteria are able to reduce elemental sulfur using hydrogen or organic substrates as electron donors [50]. However, only a few bacteria can completely oxidize acetate with sulfur reduction, including *Desulfuromonas acetoxidans *utilizing an ATP-driven succinate oxidation mechanism for acetate oxidation to CO_2 _with elemental sulfur as electron acceptor [49,51]. It seems that *P. carbinolicus *does not contain such an ATP-driven succinate oxidation mechanism, thus cannot completely oxidize acetate to CO_2 _with elemental sulfur or Fe(III) as electron donor.

### Implications

In subsurface environments, *Pelobacter *species can be closely associated with *Geobacter *species [19,20]. The *Pelobacter *and *Geobacter *metabolic models share 470 common reactions, about 70% of all reactions in the models. Genome sequence analysis of six *Geobacteraceae *family members including all four species discussed in this study suggested that the two *Pelobacter *species evolved separately with fermentative/syntrophic metabolism from a common *Geobacteraceae *ancestor with anaerobic respiratory metabolism [8]. The *P. carbinolicus *model contains reactions for fermentative growth with various organic substrates and reactions of hydrogenase and formate dehydrogenase to produce hydrogen and formate for syntrophic growth with hydrogen- and formate-consuming organisms. The *P. propionicus *model contains reactions for fermentative growth with different organic substrates producing propionate. The *Pelobacter *models contain metabolic abilities reflecting their physiological roles in the subsurface community. The evolution of the *Pelobacter *species to fermentative metabolism could also result in the loss of many *c*-type cytochromes from their genomes, since there is no need to transfer electrons outside the cells during fermentative growth, thus abolishing their abilities to directly reduce Fe(III) or the anodes of microbial fuel cells. Furthermore, the evolution of a subsurface community containing fermentative and respiratory species allowing the breakdown of complex organic substrates to CO_2 _through different species may be advantageous as each species does not need to synthesize a full set of proteins for the complete oxidization of complex organic substrates. The development of the metabolic models of these *Geobacteraceae *species will facilitate the study of their roles and interactions in the subsurface microbial community.

## Conclusions

We have developed genome-scale metabolic models of *P. carbinolicus *and *P. propionicus*. These models of *Pelobacter *metabolism can now be incorporated into the growing repertoire of genome scale models of the *Geobacteraceae *family to aid in describing the growth and activity of these organisms in anoxic environments and in the study of their roles and interactions in the subsurface microbial community.

## List of abbreviations

DCPIP: 2,6-dichlorophenolindophenol; GAM: growth-associated maintenance; gdw: gram dry weight; GPR: gene-protein-reaction; HPLC: high-pressure liquid chromatography; nGAM: non-growth associated maintenance; ORF: open reading frame.

## Authors' contributions

JS, OB, and TRF developed the genome-scale metabolic models of *P. carbinolicus *and *P. propionicus*. SAH carried out the growth experiments. JS analyzed the experimental data and drafted the manuscript. JS, SAH, and DRL conceived the study and revised the manuscript. All authors read and approved the final manuscript.

## Supplementary Material

Additional file 1 **Metabolic maps for the *P. carbinolicus *and *P. propionicus *genome-scale metabolic models**. Click here for file

Additional file 2 **All the reactions, genes, and metabolites included in the *P. carbinolicus *genome-scale metabolic model**. Click here for file

Additional file 3 **All the reactions, genes, and metabolites included in the *P. propionicus *genome-scale metabolic model**. Click here for file

Additional file 4 **All the unique reactions in each of the four *Geobacteraceae *genome-scale metabolic models**. Click here for file

## References

[B1] Narasingarao P Haggblom MM Identification of anaerobic selenate-respiring bacteria from aquatic sediments Appl Environ Microbiol 2007 73 3519 3527 10.1128/AEM.02737-06 17435005 PMC1932684

[B2] Mussmann M Ishii K Rabus R Amann R Diversity and vertical distribution of cultured and uncultured Deltaproteobacteria in an intertidal mud flat of the Wadden Sea Environmental Microbiology 2005 7 405 418 10.1111/j.1462-2920.2005.00708.x 15683401

[B3] Chauhan A Ogram A Phylogeny of acetate-utilizing microorganisms in soils along a nutrient gradient in the Florida Everglades Appl Environ Microbiol 2006 72 6837 6840 10.1128/AEM.01030-06 17021240 PMC1610308

[B4] Shimizu S Akiyama M Naganuma T Fujioka M Nako M Ishijima Y Molecular characterization of microbial communities in deep coal seam groundwater of northern Japan Geobiology 2009 5 423 433 10.1111/j.1472-4669.2007.00123.x

[B5] Dahle H Garshol F Madsen M Birkeland NK Microbial community structure analysis of produced water from a high-temperature North Sea oil-field Antonie Van Leeuwenhoek 2008 93 37 49 10.1007/s10482-007-9177-z 17588160

[B6] Schink B Fermentation of 2,3-Butanediol by Pelobacter-Carbinolicus Sp-Nov and Pelobacter-Propionicus Sp-Nov, and Evidence for Propionate Formation from C-2 Compounds Archives of Microbiology 1984 137 33 41 10.1007/BF00425804

[B7] Lonergan DJ Jenter HL Coates JD Phillips EJP Schmidt TM Lovley DR Phylogenetic analysis of dissimilatory Fe(III)-reducing bacteria Journal of Bacteriology 1996 178 2402 2408 8636045 10.1128/jb.178.8.2402-2408.1996 PMC177952

[B8] Butler JE Young ND Lovley DR Evolution from a respiratory ancestor to fill syntrophic and fermentative niches: comparative fenomics of six Geobacteraceae species BMC Genomics 2009 10 103 10.1186/1471-2164-10-103 19284579 PMC2669807

[B9] Holmes DE Nevin KP Lovley DR Comparison of 16S rRNA, nifD, recA, gyrB, rpoB and fusA genes within the family Geobacteraceae fam. nov Int J Syst Evol Microbiol 2004 54 1591 1599 10.1099/ijs.0.02958-0 15388715

[B10] Lovley DR Phillips EJ Lonergan DJ Widman PK Fe(III) and S0 reduction by Pelobacter carbinolicus Appl Environ Microbiol 1995 61 2132 2138 7793935 10.1128/aem.61.6.2132-2138.1995 PMC167486

[B11] Lovley DR Dissimilatory Fe(III) and Mn(IV) reduction Microbiol Rev 1991 55 259 287 1886521 10.1128/mr.55.2.259-287.1991 PMC372814

[B12] Haveman SA Holmes DE Ding YHR Ward JE DiDonato RJ Lovley DR c-Type Cytochromes in Pelobacter carbinolicus Appl Environ Microbiol 2006 72 6980 6985 10.1128/AEM.01128-06 16936056 PMC1636167

[B13] Butler JE Kaufmann F Coppi MV Nunez C Lovley DR MacA a diheme c-type cytochrome involved in Fe(III) reduction by Geobacter sulfurreducens Journal of Bacteriology 2004 186 4042 4045 10.1128/JB.186.12.4042-4045.2004 15175321 PMC419948

[B14] Leang C Coppi MV Lovley DR OmcB, a c-type polyheme cytochrome, involved in Fe(III) reduction in Geobacter sulfurreducens J Bacteriol 2003 185 2096 2103 10.1128/JB.185.7.2096-2103.2003 12644478 PMC151516

[B15] Lloyd JR Leang C Hodges Myerson AL Coppi MV Cuifo S Methe B Biochemical and genetic characterization of PpcA, a periplasmic c-type cytochrome in *Geobacter sulfurreducens* Biochem J 2003 369 153 161 10.1042/BJ20020597 12356333 PMC1223068

[B16] Mehta T Coppi MV Childers SE Lovley DR Outer membrane c-type cytochromes required for Fe(III) and Mn(IV) oxide reduction in Geobacter sulfurreducens Appl Environ Microbiol 2005 71 8634 8641 10.1128/AEM.71.12.8634-8641.2005 16332857 PMC1317342

[B17] Haveman SA Didonato RJ Villanueva L Shelobolina ES Postier BL Xu B Genome-wide gene expression patterns and growth requirements suggest that Pelobacter carbinolicus reduces Fe(III) indirectly via sulfide production Appl Environ Microbiol 2008 74 4277 4284 10.1128/AEM.02901-07 18515480 PMC2493185

[B18] Richter H Lanthier M Nevin KP Lovley DR Lack of Electricity Production by Pelobacter carbinolicus Indicates that the Capacity for Fe(III) Oxide Reduction does Not Necessarily Confer the Ability for Electron Transfer to Fuel Cell Anodes Appl Environ Microbiol 2007 73 5347 5353 10.1128/AEM.00804-07 17574993 PMC1950970

[B19] Holmes DE Bond DR O'Neil RA Reimers CE Tender LR Lovley DR Microbial communities associated with electrodes harvesting electricity from a variety of aquatic sediments Microb Ecol 2004 48 178 190 10.1007/s00248-003-0004-4 15546038

[B20] Vrionis HA Anderson RT Ortiz-Bernad I O'Neill KR Resch CT Peacock AD Microbiological and geochemical heterogeneity in an in situ uranium bioremediation field site Appl Environ Microbiol 2005 71 6308 6318 10.1128/AEM.71.10.6308-6318.2005 16204552 PMC1265972

[B21] Lovley DR Cleaning up with genomics: applying molecular biology to bioremediation Nat Rev Microbiol 2003 1 35 44 10.1038/nrmicro731 15040178

[B22] Mahadevan R Bond DR Butler JE Esteve-Nunez A Coppi MV Palsson BO Characterization of metabolism in the Fe(III)-reducing organism Geobacter sulfurreducens by constraint-based modeling Appl Environ Microbiol 2006 72 1558 1568 10.1128/AEM.72.2.1558-1568.2006 16461711 PMC1392927

[B23] Sun J Sayyar B Butler JE Pharkya P Fahland TR Famili I Genome-scale constraint-based modeling of Geobacter metallireducens BMC Syst Biol 2009 3 15 10.1186/1752-0509-3-15 19175927 PMC2640342

[B24] Izallalen M Mahadevan R Burgard A Postier B Didonato R Jr Sun J Geobacter sulfurreducens strain engineered for increased rates of respiration Metab Eng 2008 10 267 275 10.1016/j.ymben.2008.06.005 18644460

[B25] Segura D Mahadevan R Juarez K Lovley DR Computational and experimental analysis of redundancy in the central metabolism of Geobacter sulfurreducens PLoS Comput Biol 2008 4 e36 10.1371/journal.pcbi.0040036 18266464 PMC2233667

[B26] Butler JE He Q Nevin KP He Z Zhou J Lovley DR Genomic and microarray analysis of aromatics degradation in Geobacter metallireducens and comparison to a Geobacter isolate from a contaminated field site BMC Genomics 2007 8 180 10.1186/1471-2164-8-180 17578578 PMC1924859

[B27] Risso C Van Dien SJ Orloff A Lovley DR Coppi MV Elucidation of an alternate isoleucine biosynthesis pathway in Geobacter sulfurreducens J Bacteriol 2008 190 2266 2274 10.1128/JB.01841-07 18245290 PMC2293191

[B28] Krushkal J Yan B DiDonato LN Puljic M Nevin KP Woodard TL Genome-wide expression profiling in Geobacter sulfurreducens: identification of Fur and RpoS transcription regulatory sites in a relGsu mutant Funct Integr Genomics 2007 7 229 255 10.1007/s10142-007-0048-5 17406915

[B29] Mahadevan R Lovley DR The degree of redundancy in metabolic genes is linked to mode of metabolism Biophys J 2008 94 1216 1220 10.1529/biophysj.107.118414 17981891 PMC2212697

[B30] Smith PK Krohn RI Hermanson GT Mallia AK Gartner FH Provenzano MD Measurement of protein using bicinchoninic acid Anal Biochem 1985 150 76 85 10.1016/0003-2697(85)90442-7 3843705

[B31] Price ND Reed JL Palsson BO Genome-scale models of microbial cells: evaluating the consequences of constraints Nat Rev Microbiol 2004 2 886 897 10.1038/nrmicro1023 15494745

[B32] Price ND Papin JA Schilling CH Palsson BO Genome-scale microbial in silico models: the constraints-based approach Trends Biotechnol 2003 21 162 169 10.1016/S0167-7799(03)00030-1 12679064

[B33] Teusink B Wiersma A Molenaar D Francke C de Vos WM Siezen RJ Analysis of growth of Lactobacillus plantarum WCFS1 on a complex medium using a genome-scale metabolic model J Biol Chem 2006 281 40041 40048 10.1074/jbc.M606263200 17062565

[B34] Edwards JS Palsson BO The *Escherichia coli *MG1655 in silico metabolic genotype: its definition, characteristics, and capabilities Proc Natl Acad Sci USA 2000 97 5528 5533 10.1073/pnas.97.10.5528 10805808 PMC25862

[B35] Oh YK Palsson BO Park SM Schilling CH Mahadevan R Genome-scale reconstruction of metabolic network in bacillus subtilis based on high-throughput phenotyping and gene essentiality data J Biol Chem 2007 282 28791 28799 10.1074/jbc.M703759200 17573341

[B36] Oppermann FB Steinbuchel A Schlegel HG Utilization of Methylacetoin by the Strict Anaerobe Pelobacter-Carbinolicus and Consequences for the Catabolism of Acetoin Fems Microbiology Letters 1988 55 47 52 10.1111/j.1574-6968.1988.tb02796.x

[B37] Oppermann FB Steinbuchel A Identification and Molecular Characterization of the Aco Genes Encoding the Pelobacter-Carbinolicus Acetoin Dehydrogenase Enzyme-System Journal of Bacteriology 1994 176 469 485 8110297 10.1128/jb.176.2.469-485.1994 PMC205071

[B38] Oppermann FB Schmidt B Steinbuchel A Purification and Characterization of Acetoin-2,6-Dichlorophenolindophenol Oxidoreductase, Dihydrolipoamide Dehydrogenase, and Dihydrolipoamide Acetyltransferase of the Pelobacter-Carbinolicus Acetoin Dehydrogenase Enzyme-System Journal of Bacteriology 1991 173 757 767 1898934 10.1128/jb.173.2.757-767.1991 PMC207069

[B39] Schink B Kremer DR Hansen TA Pathway of Propionate Formation from Ethanol in Pelobacter-Propionicus Archives of Microbiology 1987 147 321 327 10.1007/BF00406127

[B40] Seeliger S Janssen PH Schink B Energetics and kinetics of lactate fermentation to acetate and propionate via methylmalonyl-CoA or acrylyl-CoA FEMS Microbiol Lett 2002 211 65 70 10.1111/j.1574-6968.2002.tb11204.x 12052552

[B41] Straub KL Schink B Ferrihydrite-dependent growth of Sulfurospirillum deleyianum through electron transfer via sulfur cycling Appl Environ Microbiol 2004 70 5744 5749 10.1128/AEM.70.10.5744-5749.2004 15466509 PMC522073

[B42] Nelson DL Cox MM Lehninger Principles of Biochemistry 2000 3 New York, NY: Worth Publishers

[B43] Laska S Lottspeich F Kletzin A Membrane-bound hydrogenase and sulfur reductase of the hyperthermophilic and acidophilic archaeon Acidianus ambivalens Microbiology 2003 149 2357 2371 10.1099/mic.0.26455-0 12949162

[B44] Ng KY Sawada R Inoue S Kamimura K Sugio T Purification and some properties of sulfur reductase from the iron-oxidizing bacterium Thiobacillus ferrooxidans NASF-1 J Biosci Bioeng 2000 90 199 203 10.1016/S1389-1723(00)80110-3 16232842

[B45] Feist AM Henry CS Reed JL Krummenacker M Joyce AR Karp PD A genome-scale metabolic reconstruction for Escherichia coli K-12 MG1655 that accounts for 1260 ORFs and thermodynamic information Mol Syst Biol 2007 3 121 10.1038/msb4100155 17593909 PMC1911197

[B46] Rubio LM Ludden PW Biosynthesis of the iron-molybdenum cofactor of nitrogenase Annu Rev Microbiol 2008 62 93 111 10.1146/annurev.micro.62.081307.162737 18429691

[B47] Hu Y Fay AW Lee CC Yoshizawa J Ribbe MW Assembly of nitrogenase MoFe protein Biochemistry 2008 47 3973 3981 10.1021/bi7025003 18314963

[B48] Methe BA Webster J Nevin K Butler J Lovley DR DNA microarray analysis of nitrogen fixation and Fe(III) reduction in Geobacter sulfurreducens Appl Environ Microbiol 2005 71 2530 2538 10.1128/AEM.71.5.2530-2538.2005 15870343 PMC1087574

[B49] Paulsen J Kroger A Thauer RK ATP driven succinate oxidation in the catabolism of *Desulfuromonas acetoxidans* Arch Microbiol 1986 144 78 83 10.1007/BF00454960

[B50] Hedderich R Klimmek O Kroger A Dirmeier R Keller M Stetter KO Anaerobic respiration with elemental sulfur and with disulfides FEMS Microbiol Rev 1999 22 353 381 10.1111/j.1574-6976.1998.tb00376.x

[B51] Gebhardt NA Thauer RK Linder D Kaulfers PM Pfennig N Mechanism of acetate oxidation to carbon dioxide with elemental sulfur in *Desulfuromonas acetoxidans* Arch Microbiol 1985 141 392 398 10.1007/BF00428855

