# Supplementary figures and images for: Constraint-based modeling analysis of the metabolism of two Pelobacter species
Source: BMC Syst Biol. 2010 Dec 23;4:174. doi: 10.1186/1752-0509-4-174 (PMC3022650; doi:10.1186/1752-0509-4-174)

## Slide 1
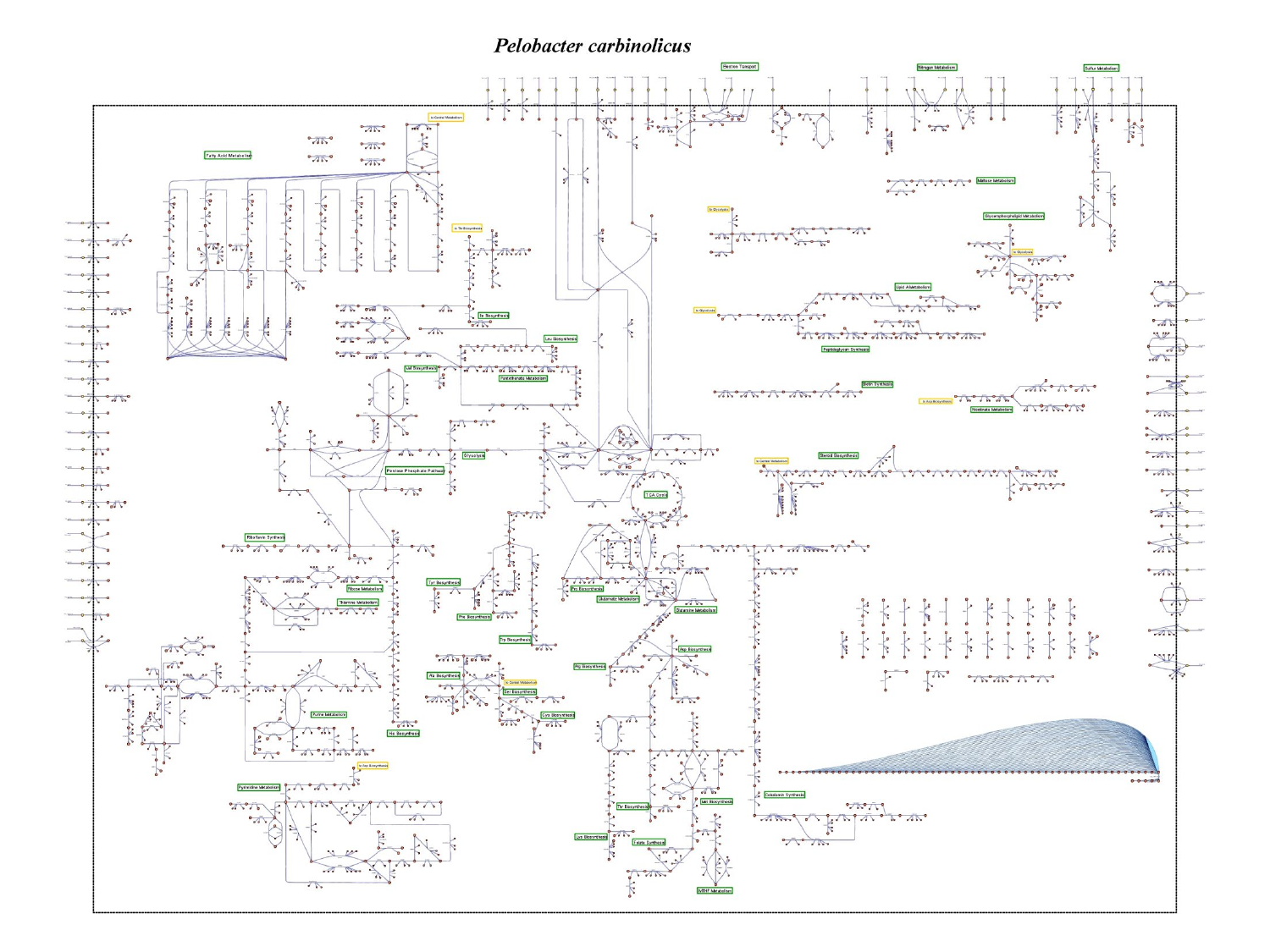

## Slide 2
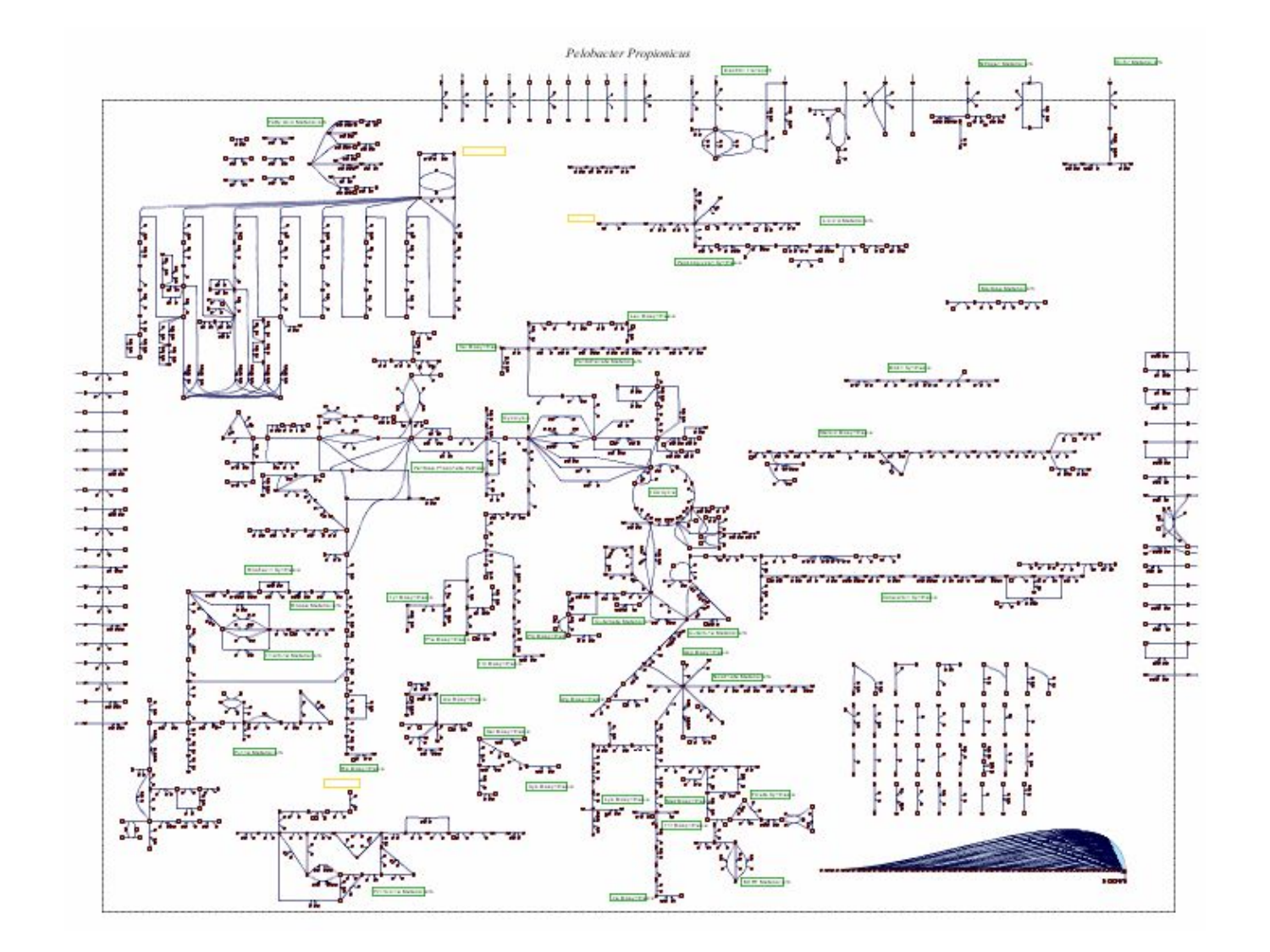

Supplement: Additional file 1 — Metabolic maps for the P. carbinolicus and P. propionicus genome-scale metabolic models. [file 1752-0509-4-174-S1.PPT]
